# The Cause of Education

**Published:** 1882-10

**Authors:** 


### The Cause of Education.

The following paper, by Dr. W. M. Dunlap, of Selma, Ala., was read before the Southern Dental Association:

Education, more especially dental education, demands the earnest consideration of this body, and holding, as I believe, the position of watchmen on the towers, to whom is entrusted the welfare of the people, may I not appeal to you, one and all, to be vigilant and give clear utterance to your views, warn the people of danger; let there be no uncertain sound given out, lest the people suffer and the sin be laid at our door.

I may ask, are we, as a profession, making the advance that we should? Are we, as a profession (in the South), coming up to the mark? If not, it would be well for us to investigate thoroughly, and ascertain the cause or causes, and when found, labor earnestly to remove them.

Going back some thirty years we find but one dental college in existence—the Baltimore College of Dental Surgery, with Chapin A. Harris, its father, at its head—striving to elevate the profession and struggling for existence. All honor to him as the pioneer in this cause. Some few good operators were found at that day, but the great majority was made up of men who adopted dentistry because they were fit for nothing else. Having failed in efforts to succeed at other things, they became dentists (so called), and from the ranks of the unsuccessful we have largely drawn our recruits ever since. With a little smattering of Harris and a limited techinal vocabulary, they set up as Sir Oracles, claiming all the knowledge, wisdom and skill to be found in the profession, pushing themselves into our associations and seeking the high places therein. And, sad to say, they, through their cheek, have succeeded in some instances in setting up as teachers. Colleges are multiplied, universities establish their dental departments, and medical institutions add dental chairs to their regular course. The result is some are found bidding for pupils, offering half rates, credit, and short terms; and when the candidate for honors signally fails, we fear that in some instances diplomas have been granted, conditional on attendance of one more term, free of charge. Such is some of the work we are compelled to face from day to day. It is surprising that colleges spring up; that, to quote from a venerable friend, "Open with prayer and close with a benediction," issuing diplomas, yet not a chair filled, nor a student to be found within its walls? Do not such irregularities in what we have been led to look upon as standard institutions of learning open the doors wide for such doings? And if so, let us find the remedy and apply it vigorously. Let us take the diploma *for only what it is worth.* Enact State laws requiring each applicant for license to stand an examination as to his fitness to practice, and let the colleges take care of themselves. Their honors are getting too cheap in too many instances, and I do think it is a mistake, and a grevious one, to adopt the plan that all restrictions as to time should be removed, and merit alone be taken. Why? Because many men get a smattering and show well that do not run well. We all know that dentists are not made in a day, and we also know that two years close application and two full courses at college is short enough time for the most talented in our midst, unless there be some upon whom a gift has been sent down. If there are any such, they themselves will proclaim it on the housetops; they do not need colleges, preceptors or any such aid; yet a cheaply earned diploma is a powerful help sometimes.

We need a high standard of qualification. We need good and true preceptors, both out of and within college doors and good and true men to take the field as co-laborers in the domain we now occupy. We should look well to the character of the men who are to be our successors. And if (as has been the case with many of us) our labors have been retarded by a scanty education and poor opportunities, we will the better do our duty by urging the rising generation to labor hard in acquiring a thorough and practical education in their profession. It is for them to supply the wants of the future, and they can best attain that end by seeking the best (though they may be the highest priced) institutions of learning.

It would be well for our associations, State, sectional and national, to adopt a rule requiring preceptors to bind their students to a full course of office instruction, and to attend college until a diploma it earned, and then leave the rest to the States to regulate the practice regardless of diplomas and I am satisfied that an earnest effort by the best men in the profession in each State in securing legislation will be crowned with success. There should be no personal ends to be accomplished in such attempts, or they will be apt either to be defeated justly or the legislation will be repulsive and speedily repealed, leaving affairs in a worse condition than at first.

[If such institutions exist as Dr. D. alludes to, he does a wrong by not pointing them out. We have many honorable institutions of learning, and they should not be brought in the range of such charges. The doctor proposes to tell what to do to remedy the matter, but the very root of the evil he does not expose.—*Ed.*]

